# Extensive Reorganization of Behavior Accompanies Ontogeny of Aggression in Male Flesh Flies

**DOI:** 10.1371/journal.pone.0093196

**Published:** 2014-04-08

**Authors:** Darrell Moore, Caleb Paquette, J. Dylan Shropshire, Edith Seier, Karl H. Joplin

**Affiliations:** 1 Department of Biological Sciences, East Tennessee State University, Johnson City, Tennessee, United States of America; 2 Department of Mathematics and Statistics, East Tennessee State University, Johnson City, Tennessee, United States of America; University of California, Los Angeles, United States of America

## Abstract

Aggression, costly in both time and energy, is often expressed by male animals in defense of valuable resources such as food or potential mates. Here we present a new insect model system for the study of aggression, the male flesh fly *Sarcophaga crassipalpis*, and ask whether there is an ontogeny of aggression that coincides with reproductive maturity. After establishing that reproductive maturity occurs by day 3 of age (post-eclosion), we examined the behavior of socially isolated males from different age cohorts (days 1, 2, 3, 4, and 6) upon introduction, in a test arena, with another male of the same age. The results show a pronounced development of aggression with age. The change from relative indifference to heightened aggression involves a profound increase in the frequency of high-intensity aggressive behaviors between days 1 and 3. Also noteworthy is an abrupt increase in the number of statistically significant transitions involving these full-contact agonistic behaviors on day 2. This elevated activity is trimmed back somewhat by day 3 and appears to maintain a stable plateau thereafter. No convincing evidence was found for escalation of aggression nor the establishment of a dominance relationship over the duration of the encounters. Despite the fact that aggressive interactions are brief, lasting only a few seconds, a major reorganization in the relative proportions of four major non-aggressive behaviors (accounting for at least 96% of the total observation time for each age cohort) accompanies the switch from low to high aggression. A series of control experiments, with single flies in the test arenas, indicates that these changes occur in the absence of the performance of aggressive behaviors. This parallel ontogeny of aggressive and non-aggressive behaviors has implications for understanding how the entire behavioral repertoire may be organized and reorganized to accommodate the needs of the organism.

## Introduction

Aggression is widespread throughout the animal kingdom [1]. Typically, male animals will defend valuable resources in the environment such as food, shelter, and access to mates. However, territorial defense is energetically costly, detracts from time available for other critical behaviors (e.g., feeding, mating, parental behavior), and often exposes the animal to greater levels of predation. It is expected that territories will not be defended unless the benefits gained from restricting resource competitors are greater than the costs of defending the resource [2], [3]. Cost-benefit studies of territoriality have been carried out on a wide variety of vertebrates, including lizards [4], salamanders [5], Siamese fighting fish [6], Golden-winged Sunbirds [7], rufous hummingbirds [8], and golden lion tamarins [9]. Such studies also have been performed on invertebrates, including aphids [10], crayfish [11], crab spiders [12], fruit flies [13], and a variety of aquatic insects [14].

In recent years, a number of invertebrate model systems have been developed in an attempt to understand the neurophysiological and genetic determinants of agonistic behaviors [15], [16], [17], [18]. Contributing to the benefits of invertebrate model systems are the ease of experimental manipulations under controlled environments, the development of detailed descriptions of behavioral repertoires, and the potential for understanding aggression at the level of individual neurons within circuits.

Among the more thoroughly investigated invertebrate model systems is the fruit fly *Drosophila melanogaster*. In nature, territorial behavior and aggression are observed in various Hawaiian *Drosophila* species that exhibit lek behavior in which males defend territories established on leaves, fern fronds, or tree limbs [19]. In *D. melanogaster*, male territoriality is seen under laboratory conditions [20], [21]. In this species, there is a positive relationship between fighting success and mating success [22]. Furthermore, territorial males have more mating success than non-territorial males [23]. The agonistic interactions performed by two males in competition for a resource containing food and a potential mate are complex, even under simplified laboratory conditions: Markov chain-based analysis of the behaviors performed during staged fights reveals that agonistic behaviors are organized as stereotyped modules [24]. Both males and females exhibit aggressive behaviors: some behaviors and behavioral sequences are found in both sexes, but others are sexually dimorphic [25]. More recent research has made significant progress in revealing the neural and genetic underpinnings of aggression. For example, the *fruitless* gene plays a major role in determining sex-specific patterns of aggression [26] as well as the establishment of male-specific neural circuits in the brain [27]. The neuromodulator octopamine is important in determining how males choose between courtship and aggression [28]. Fruit flies apparently learn from previous agonistic encounters and apply this information to establish hierarchical relationships [29]. Finally, the genetic architecture of aggression in *Drosophila* appears to possess a great deal of complexity, involving extensive pleiotropy and epistasis [30].

Similar to *Drosophila melanogaster*, male stalk-eyed flies defend valuable resources such as mates or food [31], [32], [18]. In the stalk-eyed fly *Teleopsis dalmanni*, males appear to use a sequential assessment strategy in which pairs of male flies perform ritualized, escalating agonistic behaviors that may lead to direct physical contact but do not result in injury [18].

An interesting contrast to the behavior of fruit flies and stalk-eyed flies is that of the flesh fly (*Sarcophaga crassipalpis*). Male flesh flies appear to establish territories in nature that are different from those observed in *D. melanogaster* or stalk-eyed flies. Rather than defending a well-defined resource such as food or a female fly at a particular location, males defend a space in the environment. This space presumably serves as a sentinel position, from which to detect females that may travel through the surroundings. For example, males position themselves at equidistant intervals along fence rails and roof tops and will fly from these perches to pursue passing females (K.H.J., personal observation). Analogous to what is found in nature, male flesh flies, placed in rectangular enclosures in the laboratory, show significantly lower spatial tolerance of same-sex conspecifics than do females. When placed in groups of four in circular arenas, males and females show a significant difference in the degree of clustering (measured by nearest neighbor statistics): males show a tendency towards a uniform distribution whereas females tend slightly towards a clustered distribution [33]. These findings, from experiments performed under simplified laboratory environments, suggest that males have an innate ability to maintain minimal distances among themselves that is absent in females. Females, in accord with their spatial tolerance of other females under laboratory conditions, exhibit a spatial behavior in nature that is different from that observed in males, preferring to aggregate with other carrion flies and to larviposit on carcasses already occupied with larvae [34]. In the present study, we will use the male flesh fly *S. crassipalpis* as a comparative model system for the study of aggression.

Largely absent from studies of aggression are the potential influences of ontogenetic changes in physiology and behavior. Because territorial defense and its associated agonistic behaviors are exceedingly costly, it is expected that the performance of aggressive behaviors would be restricted to situations in which the benefits exceed the costs [2]. For example, male spiny lizards coexist relatively peacefully during summer months but during the fall, when females become sexually receptive, aggression is performed at very high levels [4]. Accordingly, it would be expected that male flesh flies would be relatively tolerant of one another until the males reach reproductive maturity and the females become receptive, at which time the males would become much more aggressive.

Our first objective was to determine the age at which flesh flies become sexually mature. Once the timing of reproductive behavior was established, the goal was to test the prediction that aggressive behavior would not be expressed at high levels until the males reached reproductive age. This was accomplished by individually isolating male flies shortly after adult eclosion, holding them in isolation for a predetermined number of days, and then placing one male fly in a test arena with another male of the same age cohort. Based on video recordings of the behaviors elicited during the resulting dyadic interactions, a detailed ethogram was constructed, quantitative analyses were performed with respect to possible changes in the degree of performance of each behavior with age, and sequential analyses were used to create a behavioral transition matrix for each age cohort. The results of these analyses allowed us to address a number of fundamental questions related to the ontogeny of aggression. In contrast to the fruit fly and stalk-eyed fly model systems, we monitored all behaviors, not just those associated with agonistic interactions. Our findings show a robust ontogeny of non-aggressive behaviors as well as agonistic interactions in male flesh flies. Finally, to distinguish whether the observed changes in occurrence of non-aggressive behaviors with age were the result of the performance of aggressive acts or, alternatively, programmed to occur independently, we performed control experiments with single, isolated males.

Detailed examination of the ontogeny of aggressive behavior in flesh flies enables some insights into the organization of behavior that have not yet been pursued in other invertebrate model systems. For example, what is the process by which the organism accomplishes the ontogenetic transition from being relatively non-aggressive to aggressive? Is the transition gradual or sudden? Do aggressive behaviors exist from an early age onward or do they appear *de novo* at the appropriate time? How does the change in the performance of aggression impact the overall organizational pattern (e.g., frequencies of occurrence of each behavior, probabilities that behaviors will occur in certain stereotyped sequences, etc.) of behavior? Does the transition to the aggressive condition require a radical reorganization of behavioral patterns or just slight modifications? How much of an impact will the change in condition have on the performance of behaviors that are not involved in aggression? The answers to these questions are relevant to understanding the rules that govern the neural control of behavior and may provide insights into the relationship between consistency and plasticity [35], the compartmentalization of behavior into functional modules, and the prioritization of behaviors under different conditions.

## Materials and Methods

### Experimental Organisms

Flesh flies (*S. crassipalpis*) were maintained under nondiapause conditions (15∶9 h light:dark cycles at 25°C for all stages) in a long-term (19 years) colony at East Tennessee State University, derived from another long-term colony established about 1975 at The Ohio State University in the laboratory of Dr. David Denlinger. All of the paired male experiments in this study were conducted within an aluminum shed built in the laboratory and maintained at 24±2°C as previously described [33]. Experiments with single males were conducted in a small laboratory room kept under the same conditions.

### Age at Onset of Mating

The age at which *S. crassipalpis* began mating was determined by placing equal numbers of male and female flies at day of age 0 (the day of adult emergence) into clear glass jars (4 liter) provisioned with sugar cubes and water. The number of mating pairs in each jar was recorded once each hour throughout the photophase of the 15∶9 hour light:dark cycle through day 5. The experiment was performed at 24±2°C under low density (three females and three males per jar, 14 jars) and high density (six females and six males per jar, 15 jars) conditions. The proportion of flies mating under each density condition was determined by dividing the number of mating pairs by the total number of potential mating pairs, corrected for mortalities, at each observation time.

### Ontogeny of Aggressive Behavior in Male Flesh Flies

Male flies were collected at emergence by chilling briefly until immobilized and placed in isolation chambers (petri dish with an area 50 cm^2^). Provided *ad libitum* were sucrose (in the form of sugar cubes) and water (available from miniature microfuge tubes plugged with cotton). The isolation chambers were housed under a 12∶12 hour light:dark cycle at 24°C±2°C and visually separated from one another by black cardboard partitions. The isolation ensured that male flies were socially naïve at the time at which they subsequently were tested.

**Figure 1:**
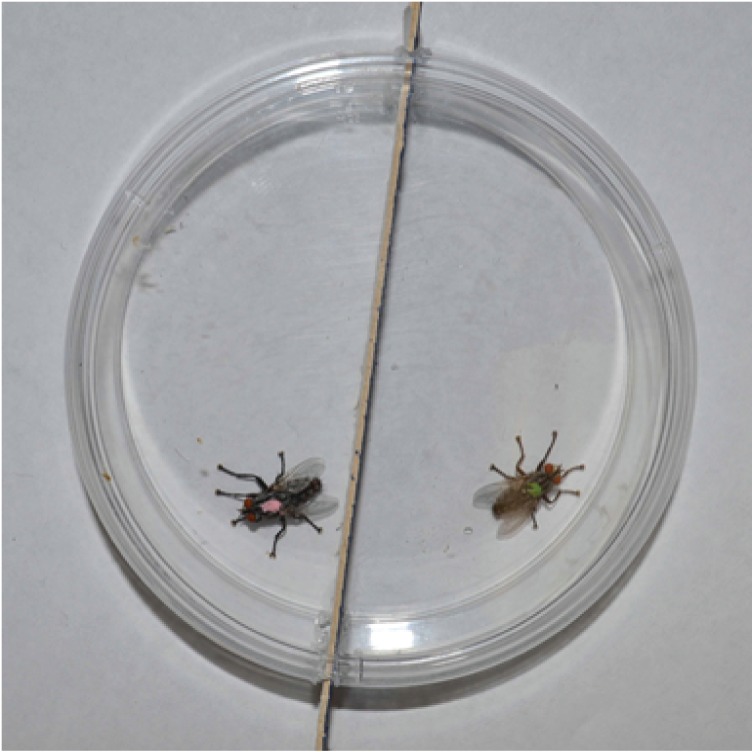
The experimental arena, viewed from above. Two male flies (each with a different color paint mark on the dorsal thorax) from the same age cohort were introduced to the arena, separated by a black partition. After 10 min, the partition was removed and observations began.

Encounters between same-age male flies were performed in a relatively simple observation arena to ascertain if the performance of aggressive behaviors is age-related. Randomly chosen, socially naïve, same-age flies were removed from their isolation chambers and individually marked on the dorsal thorax with a dot of colored enamel. Pairs of 1-, 2-, 3-, 4-, and 6-day old flies were released into a circular arena (15-cm diameter petri dish), one fly on each side of a black partition separating each half of the arena (Fig. 1). The arena did not contain food or water. There were 11 pairs tested for each age cohort with the exception of 10 pairs for day 4. Ten minutes after the flies were introduced into the arena, the partition was removed and the flies were recorded for 60 min at 30 frames/sec with a digital video camera (Sony Digital HD Handycam, HDR-UX1). Flies were used only once. Video recordings were processed with Final Cut Pro HD software (Apple Inc., Cupertino, California). Behaviors were tabulated and basic analyses were performed using JWatcher 1.0, available for public use at http://www.jwatcher.ucla.edu. To limit subjective errors, the behavioral scoring was done by a single researcher (C.P.). To facilitate (1) the construction of raster plots showing the simultaneous behavior of both members of the opponent pairs, (2) determining of the durations of the bouts of agonistic behaviors, and (3) discerning the levels of agonistic behaviors through successive 10-min intervals within the encounters, we re-coded the video recordings using the Observer XT version 11.5 (Noldus Information Technology, Wageningen, The Netherlands) using a single observer (J.D.S.).

To provide a control for the paired male experiments, individual males (using the same age cohorts under the same conditions as the paired males) were monitored in the arena. For this portion of the study, males were handled exactly as described above for the paired male experiments and their behaviors were video recorded in the same manner. However, the Observer XT version 11.5 software was used to compile and analyze the data. Also, the first and last 5 min of the 60-min recordings were not used, thus leaving 50 min of activity in each session for analyses. There were 10 males observed for each age cohort. All of the behavioral scoring of the single male data was done by a single researcher (J.D.S.).

Based on extensive video analyses, an ethogram (Table 1) was created for male flesh fly behavior exhibited in the observation arena. Behaviors were classified according to the following categories: non-interactive, interactive/non-aggressive, low-intensity aggression, and high-intensity aggression. Non-interactive behaviors included the four behaviors that occupied more time than any of the others (*standing*, *walking*, *grooming*, and *upside-down*) plus *stilt*, *bobbing*, and *jump*. All *grooming* movements were performed from a standing posture but were considered a separate behavior from *standing*. Interactive/non-aggressive behaviors were those in which the fly apparently was aware of the other fly in the arena but did not physically engage its adversary; the fly moved toward (*turn toward*, *approach*) or away from (*avoid*, *retreat*) its opponent. *Avoid* and *retreat* represented two different velocities of movement: *avoid* was relatively slow, involving locomotion *via* leg movements only whereas *retreat* was much faster, using propulsion from wing movements (Video S5). Low-intensity (*chop*, *uppercut*, *back kick*, *head butt*, *fencing*, and *boxing*) and high-intensity aggressive behaviors (*lunge*, *hold*, *wrestling*, *immobilized*) involved physical contact between the two flies. The difference between low-intensity and high-intensity aggression was characterized by limited body contact between individuals in the former and full-body contact in the latter. Because of the limited occurrence of each of the six different low-intensity aggressive behaviors, for purposes of analyses, they were combined and treated as a single behavior – *low-intensity aggression*. Also, because *immobilized* in one fly was the consequence of its opponent performing a *hold* behavior, the two behaviors always exhibited the same frequency in our analyses.

**Table 1:** Ethogram for male flesh fly behavior performed within the observation arenas.

Category	Behavior	Description
*Non-interactive*	Walking (W)	Typical locomotion throughout the arena
	Standing (Sta)	Upright, stationary, no grooming
	Grooming (G)	Upright, stationary, various grooming movements
	Upside-down (U)	On back, attempting to right itself
	Stilt (Sti)	A single extension of the legs causing the body to rise from and return to the standing position
	Bobbing (B)	Raising and lowering the body multiple times in rapid succession
	Jump (J)	Vertical leap
*Interactive/Non-aggressive*	Approach (Ap)	Fly advances within one body length of opponent
	Turn toward (T)	Fly turns to face opponent
	Avoid (Av)	Fly slowly walks away from advancing fly
	Retreat (R)	Fly quickly (using wing propulsion) moves away from advancing fly to another area of the arena
*Low-Intensity Aggression* (Lo)	Chop	Downward strike against opponent with foreleg
	Uppercut	Upward strike against opponent with foreleg
	Back Kick	Striking opponent with back leg
	Head Butt	Pushing opponent with head
	Fencing	Both flies strike each other with one foreleg
	Boxing	Both flies rear up on back legs and strike each other with both forelegs
*High-Intensity Aggression*	Lunge (L)	Fly rears up and jumps toward opponent
	Hold (H)	Grasping opponent with forelegs; opponent is immobilized
	Wrestle (Wr)	Both flies grasp each other with forelegs, strike with other legs, and spin about the enclosure
	Immobilized (I)	Being held stationary by opponent, performing struggling movements

Abbreviations for each behavior are in parentheses.

### Statistical Tests

To determine if behavioral aggression varied with age in the paired male experiments, the mean number of occurrences hour^−1^ individual^−1^ was calculated for each of eleven behaviors (see Table 1) for each age-matched cohort: *lunge*, *hold*, *wrestle*, and *low-intensity aggression* from the aggression categories; *avoid*, *retreat*, *turn toward*, and *approach* from the interactive/non-aggressive category; and *jump*, *stilt*, and *bobbing* from the non-interactive category. *Immobilized* was not included because it simply reflects the *holding* behavior of the individual’s adversary. The values were then compared across age cohorts using the Kruskal-Wallis test. Post-hoc comparisons among age cohorts, if necessary, were accomplished by Dunn’s nonparametric multiple comparisons test [36]. These statistical tests also were used to compare the durations of bouts of *wrestling* and *hold* among the different age cohorts as well as to compare the frequencies of aggressive behaviors occurring within five successive 10-min intervals during the encounters (for the day 3, 4, and 6 cohorts, combined). In all cases involving the same types of comparisons, the same statistical analyses were performed for both the paired male and single male experiments.

To determine if the four most common behaviors change with age, the amount of observation time (in minutes) occupied by the four behaviors (*standing*, *walking*, *grooming*, and *upside-down*, all from the non-interactive category) was determined for the entire age group and compared to the total observation time of the group within the arena. The resulting proportions were arcsine transformed and then evaluated across age cohorts using Tukey-type multiple comparisons [36].

Behavioral transition matrices were constructed for each age cohort by tabulating the frequencies of all changes from one behavior directly to another performed by all of the individuals in that cohort. Each resulting matrix was used to explore the existence of nonrandom associations between behaviors, assuming a Markov chain process. Because only changes in behavior were examined, the frequencies in the diagonal of each matrix were zero [37]. Likelihood ratio tests (G tests), accomplished using the CATMOD procedure in SAS (SAS Institute, Inc., Cary, North Carolina), indicated that all of the matrices showed significance (P<0.0001 in all cases); therefore, Freeman-Tukey deviates [38] were calculated for each behavioral transition within each matrix to determine which transitions occurred more often than expected by chance. The criterion for significance was set at an alpha of 0.05.

Kinematic diagrams representing the results of the transition matrix analyses were constructed for each age-cohort matrix. Within each diagram, the relative frequency of occurrence of each behavior was depicted by five different sizes of symbols (squares, circles, and triangles) in the following ascending order: <1%, 1–5%, 5–10%, 10–15%, and >15%. Significant behavioral transitions were represented by arrows connecting the symbols; the degree of departure from randomness was depicted by using three different sizes of arrows (each size also illustrated with a different color) according to the following ranges of Freeman-Tukey values, in ascending order: 1–5 (low level; black), 5–10 (intermediate level; orange), and >10 (high level; red).

## Results

### Age at Onset of Mating

**Figure 2:**
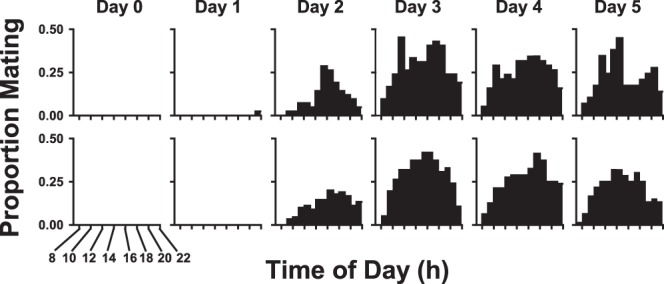
Occurrences of mating behavior with age at two density conditions. The proportion of flies mating under low (top row) and high (bottom row) density conditions with respect to adult age, in days, and time of day during the photophase of the 15∶9 hour light:dark cycle. Day 0 is the day of eclosion.

To determine the age of onset of mating behavior, equal numbers of newly emerged (day 0) male and female flesh flies were placed under high or low density conditions and then monitored for the presence of mating pairs every hour throughout the daylight hours through day 5 of age. The proportion mating (the number of mating pairs observed relative to the number of potential mating pairs) for both density conditions was essentially zero on days 0 and 1, appeared at relatively low levels on day 2, and was maintained at relatively high levels from day 3 through day 5 (Fig. 2). We conclude that mating may begin by day 2 of age but the highest proportion of flies mating occurs on day 3.

### Flesh Flies Fight

**Figure 3:**
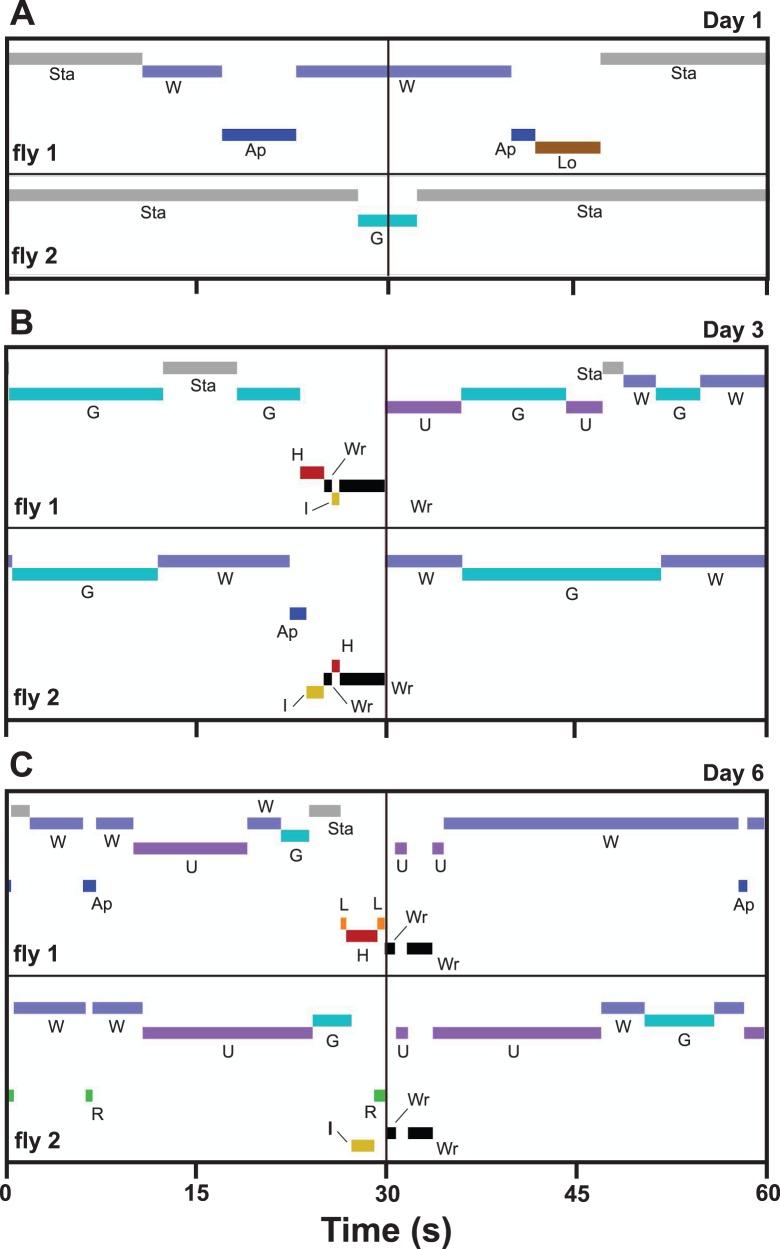
Examples of behavioral sequences and interactions observed in the experimental arena. Each plot encompasses 1-coded and also labeled (using the abbreviations in Table 1). Pairs of male flesh flies were selected from the day 1 (A), day 3 (B), and day 6 (C) age cohorts.

A raster plot (Fig. 3) showing one minute of activity for one representative opponent pair taken from three different age cohorts illustrates some general characteristics of the various behaviors performed by male flesh flies in the experimental arena. Typically, the non-interactive behaviors *walking*, *standing*, *grooming*, and *upside-down* occupied the vast majority of time during the one-hour encounters in the arena. Individual bouts of these four behaviors were several seconds to several tens of seconds in duration. All other behaviors observed in the arenas were very short in duration, from fractions of a second to several seconds. Transitions from one behavior to another were instantaneous. In the 1-min example from the day 1 age cohort (Fig. 3A), there were no high-intensity aggressive behaviors. Fly 1 exhibited mostly *standing* and *walking* behaviors whereas fly 2 performed only *standing* and *grooming*. Fly 1 transitioned from *walking* to *approach* (toward its opponent, fly 2), then resumed *walking*. It transitioned again from *walking* to *approach* and then from *approach* to *low-intensity aggression*. Neither *approach* nor *low-intensity aggression* from fly 1 yielded a change in behavior from fly 2. The flies in the day 3 example (Fig. 3B) displayed high-intensity aggressive behavior and showed a higher level of activity than those from the day 1 age cohort. For this example, *approach* performed by fly 2 was met immediately by a *hold* from fly 1. It is important to note here that when one fly performed a *hold*, its opponent was *immobilized*. This behavior was then followed in quick succession by the following sequence: *wrestling* (less than one second), *holding* (performed in this case by fly 2), a longer bout of *wrestling* (approximately 3 seconds), followed by *upside-down* in fly 1 and *walking* in fly 2. Further representing the short-duration characteristics of interactive behaviors in male flesh flies were the interactions shown in the example from the day 6 age cohort (Fig. 3C). At about 6 seconds into the record, fly 1 *approached* fly 2 which responded by *retreating* (lasting less than 1 second). Later in the record (beginning at about 27 seconds), fly 1 performed two *lunges* (each less than 0.5 second in duration), one immediately before and the other immediately after a *hold*. Just before the second *lunge*, fly 2 broke away from being *immobilized* and initiated its *retreat* (lasting less than one second). The second *lunge* from fly 1 was successful in targeting fly 2 during its *retreat* and the two opponents immediately engaged in two short bouts of *wrestling*, interrupted briefly by both flies being *upside-down*. Please see Video S1 for an example of the performance of *lunge*, Video S2 for an example of *lunge* followed by *hold*, and Video S3 for an example of *hold* followed by *wrestle*.

### Ontogeny: Interactive Behaviors

After determining the age of sexual maturity as well as the fact that male flesh flies do indeed fight each other, a primary objective was to determine if the expression of agonistic behaviors develops in concert with sexual maturation.

**Figure 4:**
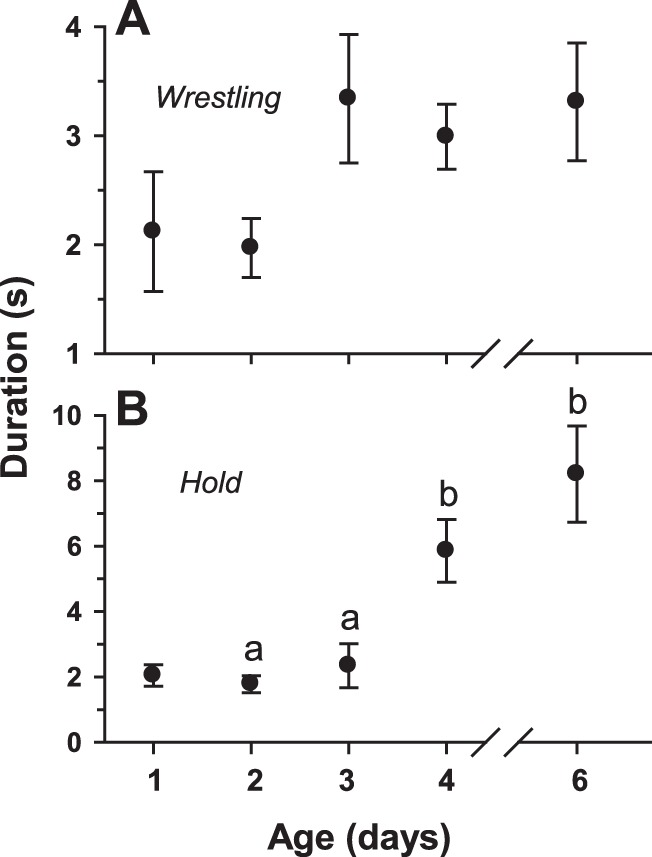
Bout durations of two high-intensity aggressive behaviors by age. (A) *Wrestling*. (B) *Hold*. Vertical bars indicate means ± s.e.m. Different letters above the bars indicate significant differences.

**Figure 5:**
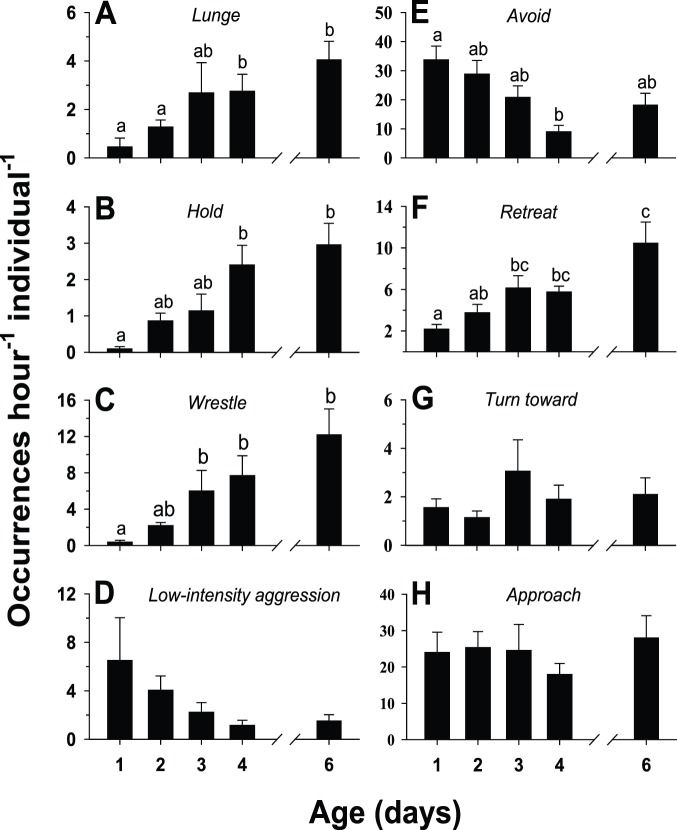
Ontogeny of interactive behaviors. Changes in the frequency of occurrence of interactive behaviors (see Table 1 for descriptions) in socially naïve, male flesh flies with respect to adult age, in days. Two individually isolated, same-age males were placed in a simple arena and the resulting behaviors were monitored for each fly. Frequencies were calculated as the number of occurrences hour^−1^ individual^−1^ for each of the following behaviors: (A) *lunge*, (B) *hold*, (C) *wrestle*, (D) *low-intensity aggression*, (E) *avoid*, (F) *retreat*, (G) *turn toward*, and (H) *approach*. Vertical bars indicate means ± s.e.m. Different letters above the bars indicate significant differences.

All of the behaviors described as interactive (see Table 1) occurred as very brief events. For example, bouts of *wrestling* (Fig. 4A) showed mean durations of only 2–3 seconds for all age cohorts while *hold* (Fig. 4B) exhibited a significant increase in duration from a mean of about 2.0 seconds in the day 1 age cohort to about 8.2 seconds in the day 6 cohort. Because of the very brief durations of these behaviors, they are reported here as occurrences/hour/individual. Most of the interactive behaviors showed a significant change in frequency with age. The behaviors associated with high intensity aggression (*lunge, hold,* and *wrestle)* all showed a significant, progressive increase in occurrence as the flies aged from day 1 to day 6 (Fig. 5A–C); in fact, relative to day 1 levels, the frequencies of occurrence of these behaviors were significantly higher by day 4 for *lunging* and *holding* and by day 3 for *wrestling*. *Low intensity aggression*, on the other hand, showed a progressive, though not statistically significant, decrease in occurrence (Fig. 5D). Two of the four interactive/non-aggressive behaviors changed significantly with age. As might be expected in parallel with the age-related increase in aggressive behaviors, *avoid* showed a significant decrease with age (Fig. 5E) whereas *retreat* exhibited a significant increase (Fig. 5F). Neither *turn toward* (Fig. 5G) nor *approach* (Fig. 5H) displayed any significant variation with age. Please see Video S4 for an example of the performance of *approach* and *avoid* and Video S5 for an example of *approach* and *retreat*.

**Figure 6:**
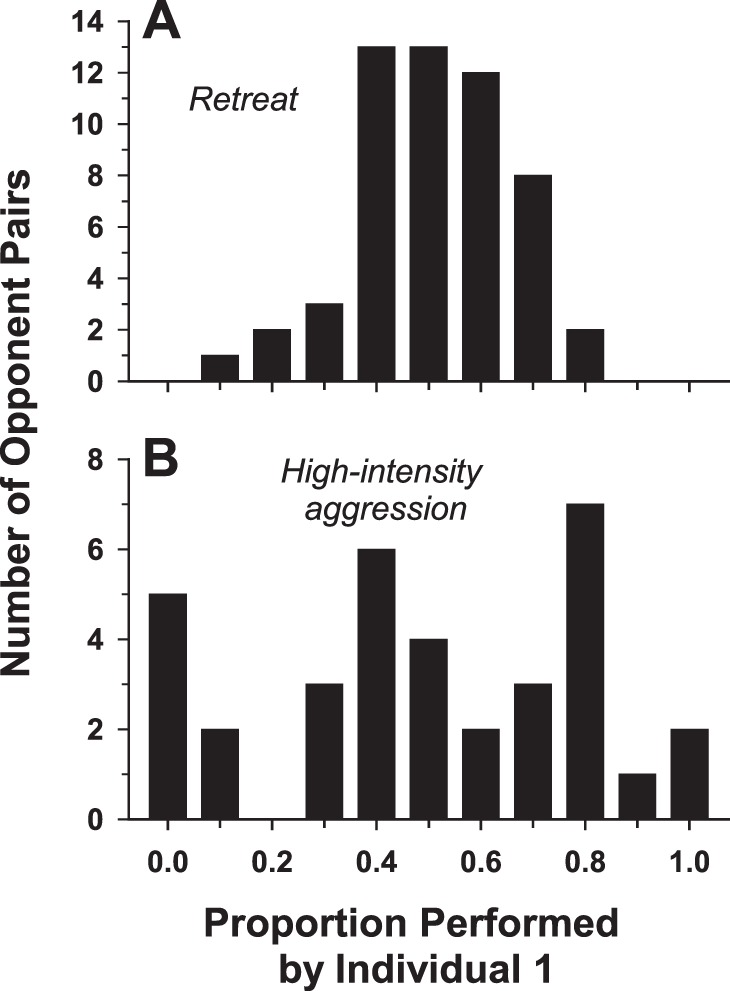
Lack of dominant individuals within the paired male encounters. Distributions of the proportions of (A) *retreat* and (B) high-intensity aggressive behaviors (*hold* and *lunge*, combined) performed by individual 1 in the paired male encounters. If the behaviors are exhibited approximately equally by both members of the pairs, the distributions should be centered around 0.5.

No convincing evidence was found for a stable dominance relationship between opponents. Using the interactive, non-aggressive behavior *retreat* as an assay for the presence of a dominant individual within pairs of opponents in the test arena, there was no case in any of the age cohorts in which only one fly exhibited the behavior. The proportion of retreats performed by the fly designated ‘individual 1′ before the observations began for each pair placed into the arena was determined for all of the pairs in all of the age cohorts. The distribution of the proportion of *retreat* behaviors performed by individual 1, calculated for each pair, was unimodal (Fig. 6A) with a mean of 0.51±0.02 (s.e.m.). Furthermore, there was no significant difference between the observed distribution and that expected for a binomial distribution (χ^2^ = 3.77, *df* = 10, *P* = 0.96) under the null hypothesis of equal probability of performance of the behavior by both members of the pair. A similar absence of domination by one fly over another was observed for high-intensity aggressive behaviors, represented by the combination of *hold* and *lunge* observed in pairs exhibiting at least two occurrences of these behaviors. Two other high-intensity aggressive behaviors were not included in this measure: *immobilized,* because it is the result of being held by the opponent fly, and *wrestling*, because both flies in the pair participate in the behavior. The distribution (Fig. 6B) of the proportion of the two high-intensity aggressive behaviors (*lunge* and *hold*) performed by individual 1 for all of the eligible pairs possessed a mean of 0.49±0.05 (s.e.m.). Although significantly different from a bimodal distribution (χ^2^ = 990.00, *df* = 10, *P*<0.0001), 60% of the eligible pairs showed ratios in the range from 0.25 through 0.75.

**Figure 7:**
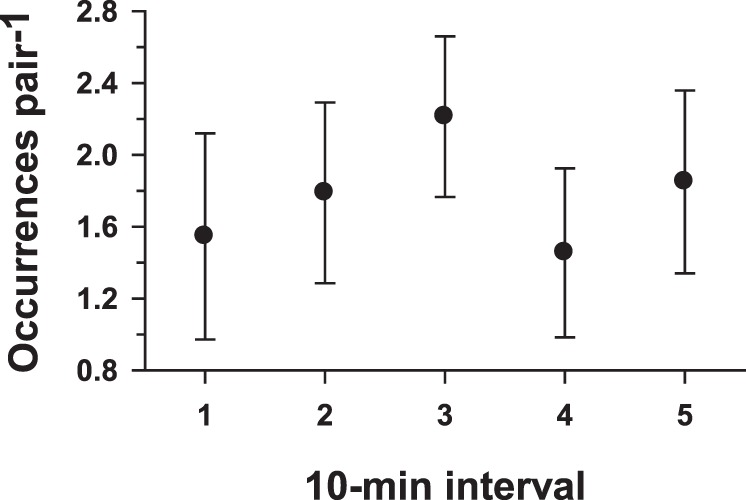
Absence of escalation of aggressive behaviors during the paired male encounters. Occurrences pair^−1^ (means ± s.e.m.) of high-intensity aggressive behaviors (*hold* and *lunge*, combined) through successive 10-min intervals within the encounters. Only the results for the day 3, 4, and 6 age cohorts are depicted because of the relative infrequency of these behaviors in the day 1 and 2 age cohorts.

Also absent in *S. crassipalpis* were any indications of escalation of agonistic behavior during the one-hour encounters. Excluding the first and last 5 min of the one-hour bouts, the number of occurrences of high intensity aggression (*hold* plus *lunge*) per pair was calculated for five consecutive 10-min intervals for each of the age cohorts (Fig. 7). No significant differences among the 10-min intervals were observed in any of the age cohorts (Kruskal-Wallis test; *P*>0.05 in all cases).

### Ontogeny: Non-interactive Behaviors

**Figure 8:**
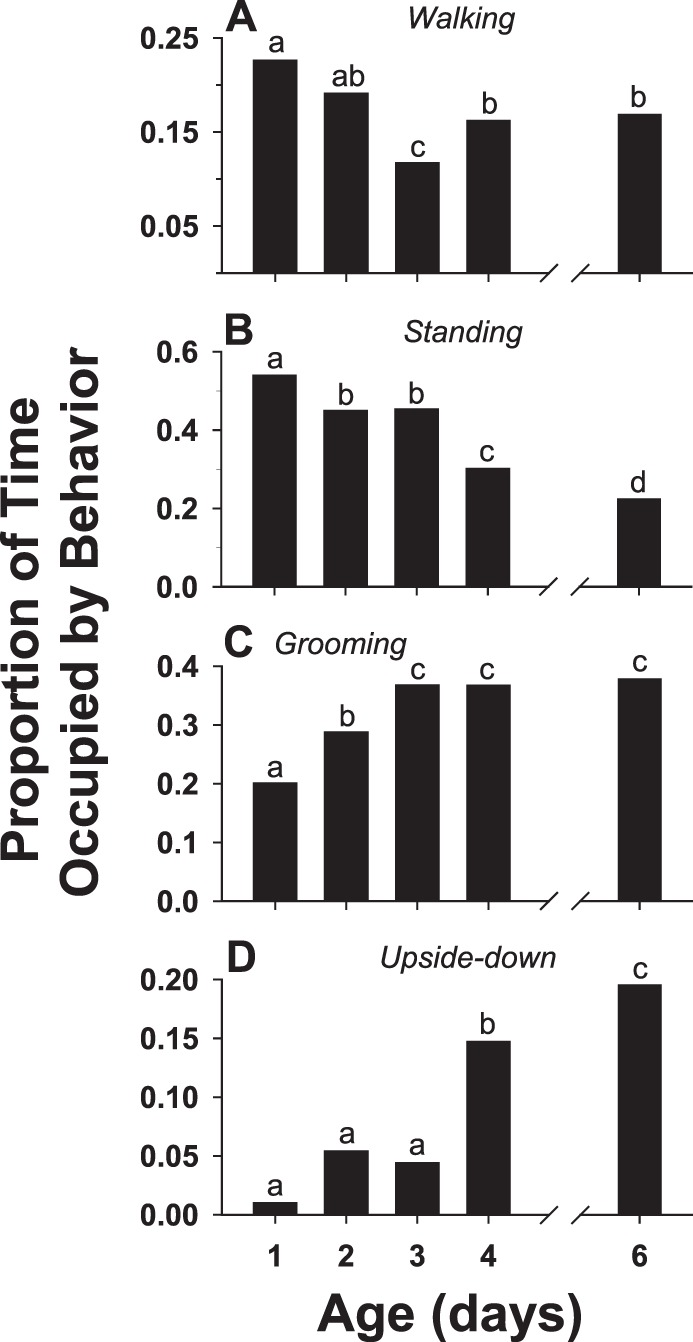
Ontogeny of predominant non-interactive behaviors. Changes in the proportions of total observation time, with respect to age (in days), occupied by each of the four most predominant non-interactive behaviors (see Table 1 for descriptions) in individual, socially naïve male flesh flies: (A) *walking*, (B) *standing*, (C) *grooming*, and (D) *upside-down*. Flies were paired in a simple observation arena (the same experiment as Fig. 5). Different letters indicate significant differences.

Four non-interactive behaviors (*walking*, *standing*, *grooming*, and *upside-down*), taken together, occupied a consistently large percentage of the time observed for all age cohorts: 97.6%, 98.1%, 98.1%, 98.0%, and 96.4% for days 1, 2, 3, 4, and 6, respectively. However, the proportions of time occupied by each of the individual behaviors exhibited significant changes with age. *Walking* (Fig. 8A) and *standing* (Fig. 8B) both showed significant declines: between days 1 and 6, the proportion of time occupied by *walking* was reduced by 25.7% and *standing* by 58.6%. *Grooming* (Fig. 8C) and *upside-down* (Fig. 8D) both exhibited significant increases: the proportion of time occupied by *grooming* increased by a factor of 1.9 and *upside-down* by a remarkable factor of 19.5 between days 1 and 6.

**Figure 9:**
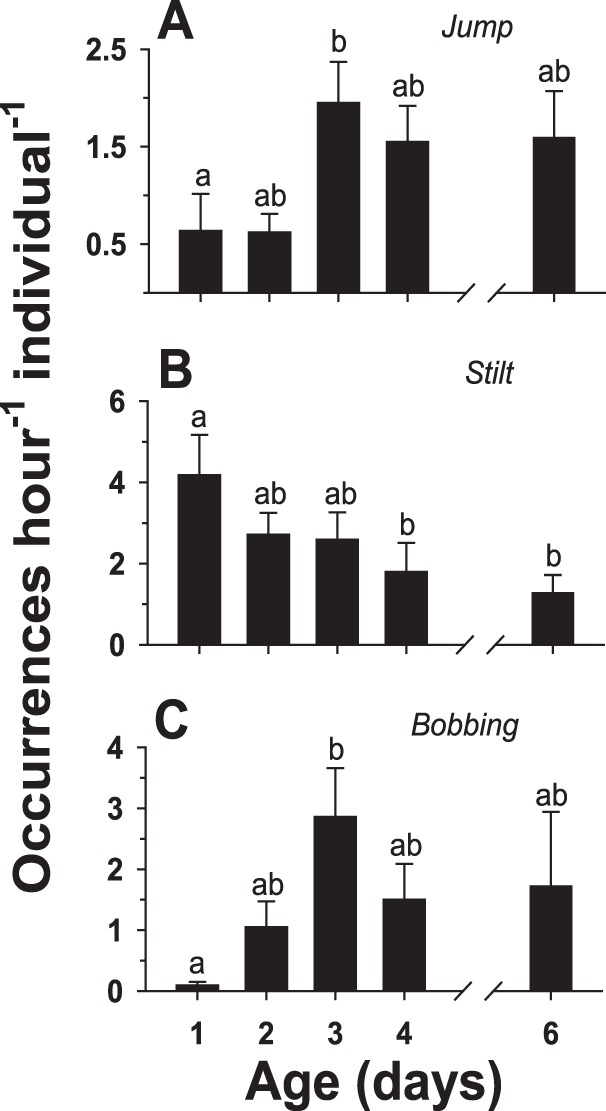
Ontogeny of infrequent non-interactive behaviors. Changes in the frequency of occurrence of three relatively infrequent non-interactive behaviors (see Table 1 for descriptions) in socially naïve, male flesh flies with respect to adult age, in days: (A) *jump*, (B) *stilt*, and (C) *bobbing*. Flies were paired in a simple observation arena (the same experiment as Figs 5–8). Vertical bars indicate means ± s.e.m. Different letters above the bars indicate significant differences.

The remaining three non-interactive behaviors also demonstrated significant changes with age. The occurrences of *jump* showed a significant increase between days 1 and 3 but declined somewhat on days 4 and 6 (Fig. 9A). *Stilt* exhibited a progressive decline in the number of occurrences per individual: relative to day 1 levels, the occurrences of *stilt* were significantly different by day 4 (Fig. 9B). Similar to the pattern exhibited by *jump*, the occurrences of *bobbing* displayed a significant increase between days 1 and 3 but declined somewhat on days 4 and 6 (Fig. 9C).

### Behavioral Transitions

To examine the temporal organization of behavior in greater detail, a behavioral transition matrix was constructed for each age-matched cohort (Table 2 depicts the transition matrix for the day 3 cohort; File S1 shows the matrices for day 1, 2, 4, and 6 cohorts). For each matrix, the behaviors were identified according to the ethogram established for male flesh flies (Table 1). We observed a total of 5075, 5990, 5098, 5203, and 7206 behavioral transitions for the day 1, 2, 3, 4, and 6 age cohorts, respectively. The first behavior in each transition was designated behavior 1 and the second was behavior 2. A log-linear model, used to compare matrices among the age-matched cohorts, showed significant interactions between behavior 1 and age (χ^2^ = 1207, *df* = 60, *P*<0.0001) and between behavior 2 and age (χ^2^ = 1214, *df* = 60, *P*<0.0001) thus revealing age-related changes in behavioral frequencies. Furthermore, the third order interaction among behavior 1, behavior 2, and age also was significant (χ^2^ = 1151, *df* = 888, *P*<0.0001), indicating that the transition probabilities were not the same among the age cohorts.

**Table 2:** Behavioral transition matrix for day 3 age cohort.

	U	B	G	I	J	T	H	Wr	Av	W	Ap	Lo	L	R	Sti	Sta	Σ
**U**	–	0	53	0	0	1	1	**44**	0	**206**	2	0	**7**	1	0	93	408
**B**	1	–	8	0	0	0	0	0	0	3	0	0	0	0	0	**51**	63
**G**	13	**50**	–	0	6	**19**	0	1	62	305	16	0	6	6	**16**	**399**	899
**I**	3	0	0	–	0	0	0	**16**	0	3	0	0	0	0	0	2	24
**J**	1	0	0	0	–	**4**	0	0	**5**	12	1	0	0	**2**	0	**16**	41
**T**	2	1	8	1	0	–	0	1	**7**	15	**13**	0	**8**	2	0	8	66
**H**	3	0	0	0	0	0	–	**16**	0	4	0	0	0	0	0	2	25
**Wr**	**33**	0	3	**8**	0	0	**7**	–	0	**50**	1	0	1	**8**	0	20	131
**Av**	**44**	0	17	1	3	**10**	0	2	–	**144**	32	**13**	**7**	**23**	3	79	378
**W**	**210**	3	322	2	9	8	0	2	22	–	**415**	1	3	18	5	233	1253
**Ap**	37	0	15	**8**	3	2	0	8	**154**	**178**	–	**16**	**10**	**36**	3	51	521
**Lo**	**6**	1	0	0	0	0	0	**4**	**11**	1	3	–	**4**	0	**2**	5	37
**L**	0	0	0	0	0	0	**17**	**31**	0	4	0	1	–	1	0	3	57
**R**	**22**	1	0	**3**	0	1	0	3	8	**60**	8	0	1	–	0	23	130
**Sti**	0	**2**	6	0	0	0	0	1	**9**	8	1	1	0	0	–	**28**	56
**Sta**	31	5	**473**	1	**20**	**21**	0	0	**100**	254	29	5	10	**33**	**27**	–	1009
**Σ**	406	63	905	24	41	66	25	129	378	1247	521	37	57	130	56	1013	5098

The matrix summarizes the frequencies at which each behavior (far left column) is followed by any other behavior (top row). Those transitions occurring more frequently than predicted by chance are indicated in bold. Descriptions of the behaviors (and their abbreviations) are summarized in Table 1.

Within each age-cohort transition matrix, Freeman-Tukey deviates [38] were calculated for each cell, enabling identification of those transitions occurring more often than predicted by chance (*P*<0.05). There were 38, 58, 55, 54, and 53 significant behavioral transitions for age cohorts 1, 2, 3, 4, and 6 days, respectively.

**Figure 10:**
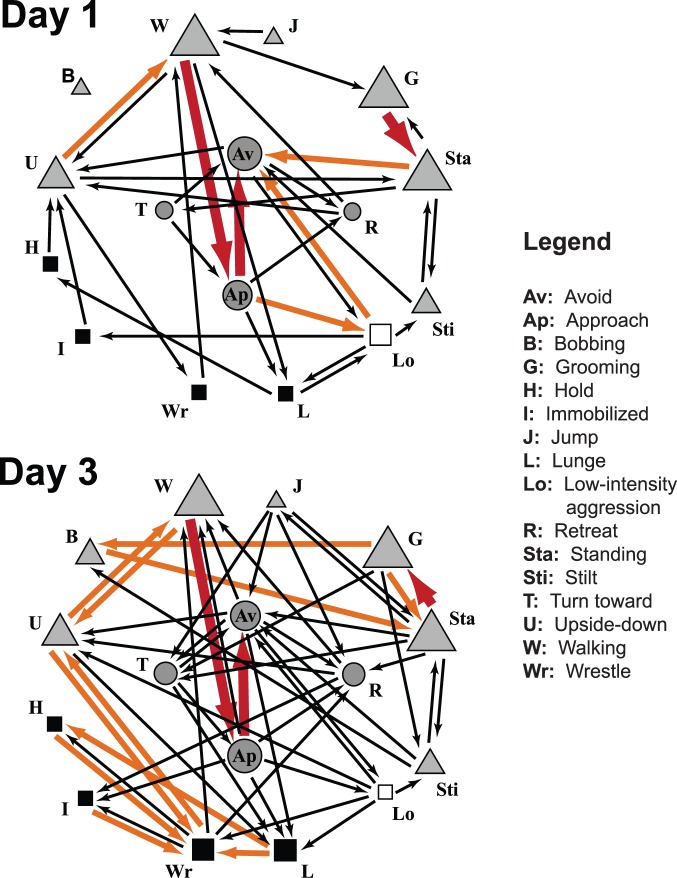
Statistically significant behavioral transitions at 1 and 3 days of age. Kinematic diagrams illustrating behavioral transitions that occurred more often than predicted by chance on days of age 1 and 3. Arrows indicate the direction of the transition. Three levels of arrow thickness, from smallest to largest, denote the degree of departure from randomness based on Freeman-Tukey values of 1–5 (black), 5–10 (orange), and >10 (red). Closed squares indicate high-intensity aggressive behaviors; open squares, low-intensity aggressive behaviors; triangles, non-interactive behaviors; circles, interactive, non-aggressive behaviors. Five sizes of symbols represent the relative frequency of occurrence of each particular behavior with respect to the entirety of behaviors for that age group: <1%, 1–5%, 5–10%, 10–15%, and >15%.

Kinematic diagrams (Fig. 10) were constructed to depict all of the significant behavioral sequences exhibited by the male flies in each age cohort. Comparisons of kinematic diagrams among the age cohorts revealed several components of behavioral organization including ontogenetic changes in behavioral sequences involving both aggressive and non-aggressive behaviors as well as behavioral sequences that were invariant with age.

Many salient aspects of behavioral organization and reorganization can be seen by comparing the kinematic diagrams for the day 1 and day 3 age cohorts (Fig. 10). Most obvious is that the day 3 cohort shows a much larger number of significant transitions and, therefore, possesses a much more complex network of sequential pathways. Many of these additional transitions involve high intensity aggressive behaviors: in the day 1 cohort, there are 10 significant transitions made to or from high intensity behaviors whereas there are 18 in the day 3 cohort. More specifically, there are only two significant transitions to the high intensity aggressive behavior *lunging* (from *approach* and *low intensity aggression*) in the day 1 cohort but there are five significant transitions to *lunging* in the day 3 cohort (from *avoid*, *turn toward*, and *upside-down* in addition to *approach* and *low intensity aggression* already present in the day 1 cohort). Similarly, there is only one significant transition to the high intensity aggressive behavior *wrestling* in the day 1 cohort (from *upside-down*) compared to five in the day 3 cohort (from *upside-down*, *immobilized*, *holding*, *lunging*, and *low-intensity aggression*). Many of the transitions involving high intensity aggression in the day 3 cohort are from one high intensity aggressive behavior to another. For example, the transitions *holding–wrestling* and *lunging–wrestling* are absent in day 1 flies but present in the day 3 cohort. Interestingly, there are a limited number of other behaviors that lead to one or more of the high intensity aggressive behaviors. These are *low intensity aggression*, *approach*, and *upside-down* in the day 1 cohort. The same three pathways to high intensity aggression are present in the day 3 cohort, with the additions of *turn toward*, *avoid*, and *retreat*.

Not all changes in behavioral transitions between the day 1 and day 3 cohorts involve aggressive behaviors (Fig. 10). Two notable examples involve *bobbing* and *jump*. *Bobbing* contributes no significant behavioral transitions in the day 1 cohort but is paired with *grooming*, *standing*, and *stilt* in the day 3 flies. *Jump* is paired only with *walking* in day 1 flies but this transition is lost in the day 3 cohort and is replaced by five different associations.

A number of behavioral transitions are invariant between the day 1 and day 3 cohorts. Most noticeable are the *walking–approach*, *approach–avoid*, and *grooming–standing* transitions, all of which involve behaviors occurring at high frequency and transitions exhibiting a high degree of departure from randomness (Fig. 10).

**Figure 11:**
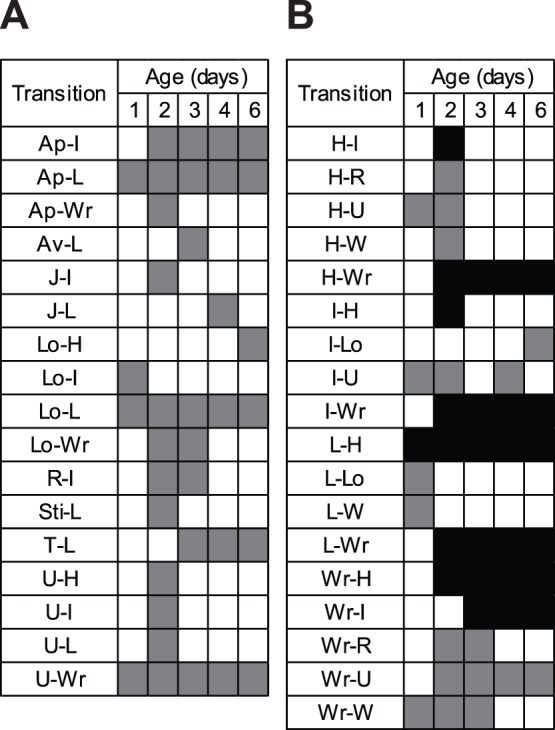
Behavioral transitions involving high-intensity aggressive behaviors by age. All transitions are depicted as first behavior followed by the second. (A) Transitions to high-intensity aggressive behaviors from other behaviors. (B) Transitions from high-intensity behaviors to other behaviors. Gray cells indicate the presence of significant transitions in which only one of the behaviors is a high-intensity aggressive behavior. Black cells indicate significant transitions in which both behaviors are high-intensity aggressive.

The radical differences between relatively non-aggressive males at day 1 of age and the much more aggressive males at day 3 were achieved by an abrupt increase in the involvement of high-intensity aggressive behaviors on day 2. There were only three behaviors that directly preceded high-intensity aggressive behaviors on day 1 but this number escalated to 6, 6, 5, and 5 on days 2, 3, 4, and 6, respectively (Fig. 11A). Also appearing abruptly on day 2 was an increase in the number of significant transitions from one high-intensity aggressive behavior to another: only 1 appeared on day 1 but there were 7, 6, 6, and 6 of these exclusively high-intensity aggressive transitions on days 2, 3, 4, and 6, respectively (Fig. 11B). Based on transitions occurring more often than expected by chance over all of the age cohorts, 8 different behaviors served as pathways leading to high-intensity aggressive behaviors (Fig. 11A) and 4 participated as exits away from high-intensity aggression (Fig. 11B).

**Figure 12:**
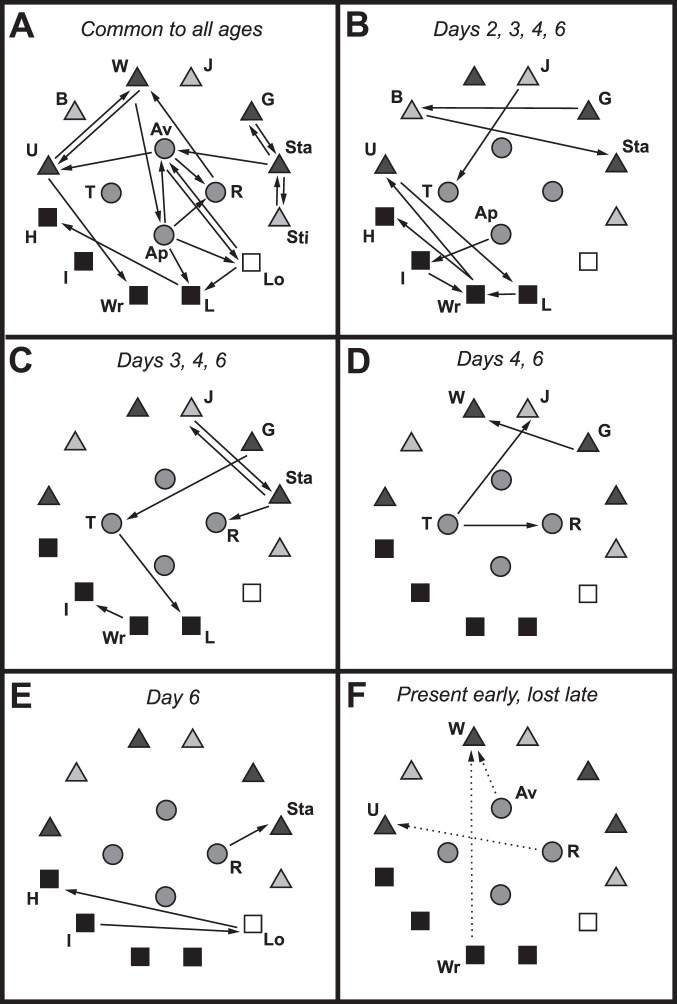
Summary of progressive changes in behavioral transitions with age. Simplified kinematic diagrams illustrating trends associated with the progressive ontogeny of aggressive behavior in male flesh flies. Symbols indicate the particular behaviors and arrows the direction of the transitions that occurred more often than predicted by chance, as in Fig. 6; abbreviations as in Table 1. (A) Behavioral transitions common to all age cohorts. (B) Transitions not present on day 1 of age, but existing from day 2 onwards. (C) Transitions absent on days 1 and 2, but present from day 3 onwards. (D) Transitions not present on days 1, 2, and 3, but existing on days 4 and 6. (E) Transitions present on day 6 but not observed prior to that. (F) Three transitions that were eliminated, rather than added. Av-W and Wr-W were present on days 1, 2, and 3, but absent on days 4 and 6. R-U was present on days 1–4 but absent on day 6.

Rather than comparing the rather complex kinematic diagrams for each age cohort with one another, the progression of age-related changes in behavioral transitions is summarized graphically in a series of simplified kinematic diagrams (Fig. 12). There were 20 significant (i.e., occurred more often than expected by chance) transitions common to all age cohorts (Fig. 12A). Among these transitions, four were associated with high intensity aggressive behaviors. Each succeeding day of age was accompanied by additional significant transitions that were not present during any of the preceding days. For example, eight more significant transitions appeared by day 2 of age and were present in all of the older age cohorts (Fig. 12B): five of these involved high intensity aggression. Another five significant transitions were absent on days 1 and 2, but were present on all days thereafter (Fig. 12C): two involved high intensity aggressive behaviors. Another four significant transitions were absent on days 1, 2, and 3 but present on days 4 and 6; none of these transitions were associated with high intensity aggression (Fig. 12D). Finally, three significant transitions appeared on day 6 but were absent in all of the younger age cohorts (Fig. 12E): two of these involved high intensity aggressive behaviors. Only three significant transitions were eliminated throughout the observed age progression (Fig. 12F): two were present on days 1, 2, and 3 but absent on days 4 and 6 (including one transition involving high intensity aggression) and one was present on days 1 through 4 but absent on day 6. In addition to the behavioral transitions summarized above, there were a number of transitions that were not classified as contributing to consistent, progressive changes with age (Table 3). Most notable were the large number of significant behavioral transitions that occurred only on day 2 or only on day 3.

**Table 3:** Statistically significant behavioral transitions not obviously associated with ontogeny of aggression.

Age of occurrence (days)	Transitions
1	J-W,L-W
2	Ap-R, Av-B, Av-J, Av-Sti, H-I, H-R, H-W, I-H, J-B, J-I, R-B, Sti-L,T-Lo, U-H, U-I
3	Ap-W, Av-L, G-Sti, J-R, Lo-U, Sti-B
4	Ap-Sti, J-L, J-U, R-J
1, 3	Lo-Sti
1, 4	L-Lo
2, 3	Ap-Wr, J-Av, R-I
2, 4	Sti-Lo
1, 2, 4	I-U
1, 2, 6	H-U
1, 3, 4	Sti-Av
1, 4, 6	W-G, U-Sta
2, 3, 6	Wr-R
1, 2, 3, 6	Av-T, T-Ap
1, 3, 4, 6	Sta-T

Behavioral transitions are represented as pairs (behavior 1 followed by behavior 2); abbreviations and descriptions of the behaviors as in Table 1.

### Ontogeny of Behavior in Single Males

**Figure 13:**
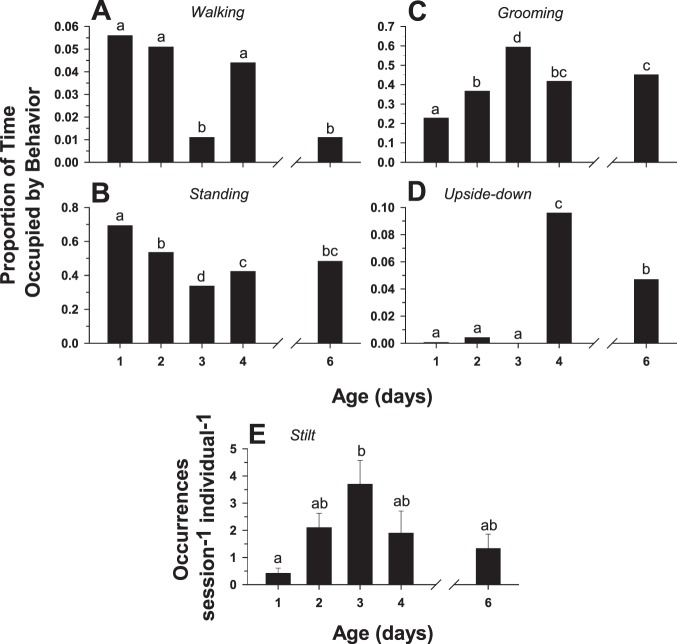
Ontogeny of behavior in the single male flies. Changes in the proportions of total observation time, with respect to age (in days), occupied by each of the four most predominant behaviors (see Table 1 for descriptions) in the individual fly experiments (A) *walking*, (B) *standing*, (C) *grooming*, and (D) *upside-down*. Also shown are changes in the frequency of occurrence of the behavior *stilt* (E), calculated as the number of occurrences hour^−1^ individual^−1^. Vertical bars indicate means ± s.e.m. Different letters above the bars indicate significant differences.

To determine whether the significant changes with age in the performance of non-aggressive behaviors observed in opponent pairs of male flesh flies occur as a result of the expression of aggressive behaviors or, alternatively, occur as autonomous behavioral developments, a control experiment with isolated single males was carried out. With respect to the proportion of time occupied by the behavior, *walking* and *standing* showed significant decreases while *grooming* and *upside-down* exhibited significant increases with age in single males (Fig. 13A–D). These findings parallel the results from the opponent pair experiments and indicate that the ontogenetic changes in these non-aggressive behaviors are not contingent upon the performance of agonistic behavior. In contrast to observations in opponent pairs, occurrences of *stilt* did not show a steady decline with age but rather increased between days 1 and 3 (Fig. 13E). Also contrary to the results in the opponent pairs, there were no instances of the behavior *bobbing* observed in any of the single male age cohorts and the behavior *jump* was exceedingly rare, occurring only twice in the day 2 cohort and once in the day 3 cohort.

**Figure 14:**
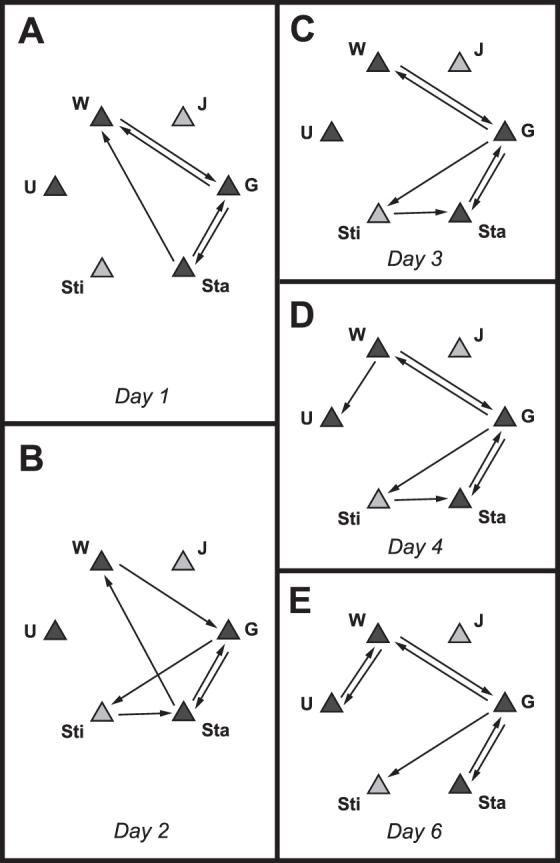
Statistically significant behavioral transitions for each of the age cohorts in the single male experiments. Simplified kinematic diagrams illustrating behavioral transitions that occurred more often than predicted by chance on days of age 1 (A), 2 (B), 3 (C), 4 (D), and 6 (E). Arrows indicate the direction of the transition. Abbreviations refer to the behaviors (see Table 1).

The rather limited behavioral repertoire performed by single flies nevertheless showed some significant transitions common to all age cohorts and other transitions that changed with age (Fig. 14). The *walking–grooming* transition was significant for all age cohorts and the *grooming–walking* transition was significant in all but the day 2 age cohort. Also significant in all age cohorts were the *grooming–standing* and the *standing–grooming* transitions. Significant transitions varying with age were (1) *standing–grooming*, present on days 1 and 2 but absent thereafter, (2) *grooming–stilt*, absent on day 1 but present thereafter, (3) *stilt–standing*, absent on days 1 and 6 but present on days 2, 3, and 4, (4) *walking–upside-down*, absent on days 1, 2, and 3 but present on days 4 and 6, and (5) *upside-down–walking* which appeared only on day 6. There were no significant transitions involving the behavior *jump* in any of the age cohorts.

## Discussion

Many gaps exist in our understanding about the neural control of behavior in animals [39], [40]. In the present study, we introduce the flesh fly *S. crassipalpis* as a model system for exploring fundamental questions concerning how the nervous system may organize a variety of behaviors, including aggressive acts, and how these patterns may change with age. After establishing a high-resolution ethogram, we investigated the ontogeny of aggressive behavior, an aspect of aggression that has received limited attention. For this study, individual flies were kept isolated from one another beginning shortly after eclosion until the age at which they were tested in a minimal arena, thereby excluding most potential external influences on behavior (social interactions, encounters with predators, changes in food availability, the presence of a potential mate, etc.) except that of light:dark cycles and chronological age. In contrast with other model systems, our observations were not limited to agonistic actions but encompassed non-aggressive behaviors as well, providing insights into how aggressive behaviors are integrated with other behavioral programs.

Satisfying our first objective, we discovered that mating in *S. crassipalpis* begins as early as day 2 of age but most flies exhibit mating behavior by day 3 (Fig. 2). This finding is consistent with observations of another species of flesh fly *Neobellieria bullata* in which males were not successful at capturing females until 2 days after eclosion [41].

Based on the determination that sexual maturation occurs by approximately day 3 of age, we tested the premise that aggressive behavior in this species would not be expressed fully until the age of sexual maturity. The evidence supporting such an ontogeny of aggression was that all four high-intensity aggressive behaviors (*lunge, wrestle, hold,* and *immobilized*) showed a significant, progressive increase in frequency with age (Fig. 5A–C). By day 3 or day 4 of age, all of these behaviors occurred at significantly higher levels relative to levels observed for flies at day 1 of age. On the other hand, low-intensity aggressive behaviors exhibited a progressive, though not significant, decline in frequency with age (Fig. 5D).

Age-related changes in aggression certainly occur in many other insects. In *D. melanogaster*, for instance, fighting is not observed on the first day after emergence as adults, but can be demonstrated reliably by day 3 [20], [22], [13], [24]. In a study examining the influence of age on territorial behavior, Hoffman [42] filmed groups of six newly eclosed *D. melanogaster* males that were caged with three 1-day old virgin females for 32 consecutive hours and found that males were first observed courting females about 9.5 hours after introduction to the cage and the first mating occurred about 3.9 hours later. The first instances of lunging were observed about 12 hours after mating, followed about 2 hours later by the first instances of territorial defense of a food source. However, because of the continuous presence of other males and females in these experiments, accumulated experience with conspecifics cannot be differentiated from age as a contributor to the expression and timing of agonistic behaviors. Nevertheless, these results plus other findings from the same study [42] (e.g. older males establish territories more readily, are more successful at holding territories, and escalate agonistic interactions more readily than younger males) indicate that age is a major determinant in the expression of agonistic behavior in *D. melanogaster*. For the present study, in an effort to separate the influence of age from social interactions, the flies were kept isolated from all other flies until the time of the 1-hour encounter with an opponent of the same age.

The mechanisms underlying the age-related increases in aggressive behaviors in the flesh fly are unknown. There may, however, be some insights from gene expression patterns in *Drosophila*. For example, Ruedi and Hughes [43] showed that many genes associated with courtship behavior in *D. melanogaster* males were expressed statically, and some dynamically, with changes in age but not with social experience (exposure to females). In another study, adult *D. melanogaster* males given a brief exposure to females failed to show courtship gene expression changes relative to naïve adult males [44]. On the other hand, Ellis and Carney [45] found 16 genes that change expression when males court females as well as 240 that were specific to male-male interactions. Assuming that courtship and territorial (aggressive) behaviors go hand-in-hand, these studies suggest a fundamental ontogenetic program operating independently of environmental influences as well as a substantial set of genes that are responsive to social interactions.

The ontogeny of agonistic behavior in male *S. crassipalpis* apparently is based upon major increases in the frequency of behaviors involving full-body contact (high-intensity aggressive behaviors) whereas those behaviors characterized as *low-intensity aggression* (a composite of 6 different behaviors, see Table 1), involving minimal contact (primarily striking or pushing with one or two legs), do not increase in frequency with age. Before sexual maturity (i.e., day 1), *low-intensity aggression* occurred more frequently than all of the high-intensity aggressive behaviors combined. By the age of sexual maturity (day 3), however, *low-intensity aggression* declined to less than half of its previous level and occurred less frequently than either of the individual high-intensity behaviors *lunge* and *wrestle*. These results suggest that *low-intensity aggression* does not play a major role in agonistic interactions in *S. crassipalpis*. Further underscoring this point is the finding that several statistically significant transitions involving other behaviors serve as pathways to or from the high-intensity aggressive behaviors (Fig. 11). All of these occur at relatively low frequencies (black arrows in Fig. 10) with the exception of the *upside-down–wrestling* transition on days 2, 3, 4, and 6 and *wrestling–upside-down* on days 3, 4, and 6. Also, a large number of transitions to high-intensity aggressive behaviors, although not individually reaching statistical significance, are shared among a variety of other behaviors (Table 2, File S1) including *walking*, *standing*, and *grooming*. The preeminence of high-intensity aggressive behaviors appears in stark contrast to the situations in both *D. melanogaster* [24] and *T. dalmanni* [18] in which high-intensity behaviors occur less frequently than low-intensity behaviors during dyadic contests between males. These differences in the structure of agonistic behaviors between flesh flies, on the one hand, and fruit flies and stalk-eyed flies on the other, may reflect fundamental differences in mating systems. For instance, flesh flies employ a sit-and-wait strategy in which males occupy lookout positions and chase and mate with females that fly within sight-range. In comparison, *D. melanogaster* and *T. dalmanni* defend spatially constrained resources such as females or food territories and appear to assess the relative strength of their rivals through behavioral sequences consisting mostly of low-intensity aggressive behaviors that may escalate to high-intensity interactions but often end before reaching that stage.

Several findings in this study are not consistent with the formation of stable dominance relationships between *S. crassipalpis* males. First, male flesh flies apparently do not assess an opponent through sequences of low-intensity agonistic interactions. In contrast, *S. crassipalpis* males exhibit low-intensity aggressive behaviors (Fig. 5D) at a lower frequency than high-intensity aggressive behaviors. Next, the distributions of the occurrences of *retreat* (Fig. 6A) or high-intensity aggression (Fig. 6B) by individual members of the opponent pairs indicate that, in most pairs, these behaviors are not performed predominantly by one member of the pair. Finally, there is no escalation of high-intensity aggressive behavior during the one-hour encounters in the arena (Fig. 7).

The sit-and-wait strategy of male flesh flies in nature may share some characteristics with territorial defense behaviors observed in male speckled wood butterflies (*Parage aegeria*) [46]. In this species, males occupy spots of sunlight on the woodland floor where they perch on prominent features of the vegetation. From these perches, they fly out to inspect passing objects, including females. These sunspot territories are contested by males and, although the manner by which the winners are decided is not understood, recent experiments suggest that more intrinsically aggressive males become sunspot residents and that previous wins reinforce the male’s ability to take over territories from other males [47]. Our experiments with flesh flies were conducted under simplified, artificial conditions and were designed primarily to discern if the intrinsic motivation to perform aggressive behaviors changes with age. The absence of detailed field observations of *S. crassipalpis* behavior limits our ability to interpret our findings with respect to natural conditions. However, the lack of escalation as well as the absence of dominant individuals during the encounters in the arena are contrary to findings in *D. melanogaster* [24], stalk-eyed flies [18], and butterflies [46], [47] and suggest some testable hypotheses. For example, it is possible that the extended social isolation imposed on our flies before staging the encounters in the arena may prevent them from acquiring some differential experience necessary to determine dominance. Alternatively, perhaps the 1-hour encounter duration, under the conditions of our experiments, is not long enough to establish a dominant individual. Yet another alternative is that dominance may be determined by territorial residency status [46] in nature but the artificial conditions in the arena are not sufficient for the establishment of residency. Perhaps relevant to the establishment of a territory is the amount of available space in the environment, an aspect that could be explored in the laboratory by varying the area in our test arenas. Finally, one intriguing possibility is that there may be no true high-intensity aggression at all in *S. crassipalpis.* This scenario would be consistent with the finding that dominance relationships and fighting escalation did not occur during the encounters but difficult to reconcile with the apparent ferocity of the fights (Videos S1, S2, and S3) and the significant increases in levels of interactive behaviors with age (Fig. 5).

One of the basic questions concerning the ontogeny of aggression in the present study was how changes in aggressive behaviors might affect the performance of non-aggressive behaviors. In the case of interactive, non-aggressive behaviors, two changed significantly with age (*avoid* decreased and *retreat* increased in frequency) while two others (*turn toward* and *approach*) showed no significant variation with age (Fig. 5E–H). All of the seven remaining behaviors, all classified as non-interactive, demonstrated significant changes with age (Figs 8, 9). Interestingly, for all age cohorts, the vast majority of time (between 96.4% and 98.1%) during the observations was occupied by just four of these behaviors: *standing*, *walking*, *grooming*, and *upside-down*. However, the amount of time spent on these four behaviors relative to each other exhibited a radical reorganization with age: *walking* and *standing* decreased significantly whereas *grooming* and *upside-down* increased significantly (Fig. 8). The finding that many behaviors, especially non-interactive behaviors, show substantial changes in performance in parallel with the ontogeny of aggressive behaviors is unexpected: each agonistic interaction is very brief, typically lasting only a few seconds (Figs. 3,4), whereas the non-interactive behaviors occupy the vast majority of the fly’s time during the observations. The results from the single male experiments (Fig. 13) show that the same age-related increases (*grooming, upside-down*) and decreases (*walking, standing*) in the amount of time occupied by these non-interactive behaviors occur in the absence of an opponent. Therefore, the reapportioning of the non-aggressive behaviors relative to one another with age does not require the performance of aggressive behaviors. These findings suggest interesting hypotheses concerning the organization of behavior. For example, aggressive and non-aggressive behaviors may be connected functionally to one another by common control circuits such that the levels of *standing*, *walking*, *grooming*, and *upside-down* are related to thresholds for the release of aggressive behaviors. Alternatively, the aggressive and non-aggressive behaviors may have no functional connections or common modulatory controls but their age-related changes simply may be programmed to occur simultaneously. This aspect of behavioral organization has received little study in any model system and deserves further examination. One possible consequence of these findings is that experimental manipulation of aggression levels (e.g., through neurohormonal or neurogenetic treatments) may have collateral effects on very disparate behavioral programs.

The robust age-related changes observed in *grooming* and *upside-down* warrant speculation concerning their potential relevance to the ontogeny of aggression in male flesh flies. Our observations revealed that, in the experiments with paired males, the amount of time devoted to *grooming* nearly doubled from about 20% on day 1 to about 37% by day 3 and maintained this high level of activity on days 4 and 6 (Fig. 8C). The same trend was observed in the experiments with single males (Fig. 13C). Grooming in insects encompasses a suite of stereotyped movements designed to remove debris and pathogens from body surfaces and has been shown to improve olfactory acuity of the antennae [48]. Many insects methodically self-groom, even in the absence of pathogens and debris [48], [49]. The remarkable increase in time invested in *grooming* among male flesh flies in our study (both with and without opponents) perhaps reflects an age-related up-regulation of an internal program to ensure that sensory organs are clear of obstructions in preparation for agonistic interactions and mating. Exhibiting an even greater change was the amount of time spent in the behavior *upside-down* in the paired male experiments, increasing from 1% on day 1 to about 4% on day 3 and nearly 20% on day 6. This extraordinary escalation is not understood but possibly may be the result of a general, heightened state of arousal associated with the increase in aggressive tendencies or an increase in failed attempts to escape the confines of the arena. The finding that upside-down also increases with age in isolated males (Fig. 13D) supports the idea that age-related changes in this behavior are only partially in response to activity in the opponent fly. Further exploration of *grooming* and *upside-down* may yield insights into the interactions between circuits driving aggression and those controlling non-aggressive behaviors.

Rather than appearing *de novo* in parallel with reproductive maturity, aggressive behaviors exist from an early age in male *S. crassipalpis*. However, as the flies progress from the relatively non-aggressive condition to the aggressive condition (i.e., day 1 to day 3 of age), there are pronounced increases in both the frequency of occurrence of high-intensity aggressive acts (Fig. 5A–C) and the number of statistically significant transitions involving high-intensity aggression (Figs 10–12). With respect to behavioral transitions, the transformation to the aggressive condition is abrupt: there are 10 statistically significant transitions involving high-intensity aggression on day 1 of age but the number escalates to 26 on day 2 and then declines somewhat to a plateau of 18, 16, and 18 on days 3, 4, and 6, respectively (Fig. 11). A large majority (13) of these behavioral transitions are shared in common among days 3, 4, and 6, indicating establishment of a stable organization of temporal patterns by the age of sexual maturation. The steep increase in transitions involving high-intensity aggressive behaviors on day 2 followed thereafter by a lower, stable number is somewhat reminiscent of the typical pattern of development in nervous systems in which there is an over-production of axonal projections and synaptic connections followed by a pruning back to numbers and patterns seen in adults [50]. This possibility presumably could be approached by comparing neuropil volumes and dendritic morphologies [51] in the brains of males from different age cohorts.

In common with the well-established insect model systems *D. melanogaster* and *T. dalmanni*, *S. crassipalpis* males possess a highly structured repertoire of behavioral transitions. However, in contrast to these two other insect model systems, there were relatively few statistically significant transitions between low- and high-intensity aggressive behaviors. Within any age cohort, whether before (days 1 and 2) or after the age of sexual maturity (days 3, 4, and 6), *low-intensity aggression* exhibited statistically significant transitions only to two high-intensity aggressive behaviors at relatively low frequencies (denoted by thin, black arrows in Fig. 10). These results, in concert with the relatively infrequent occurrence of low-intensity aggression on days 3, 4, and 6 (Fig. 5D), suggest the absence of a strategy (present in *D. melanogaster* and *T. dalmanni*) in which the flies progress through low-intensity aggressive behaviors in order to assess the strength of the opponent and avoid high-intensity agonistic interactions. Instead, after sexual maturity, *S. crassipalpis* males readily engage in high-intensity aggressive behaviors. In fact, flesh flies transition from one high-intensity behavior to another at relatively high frequencies (orange arrows in Fig. 10).

The utility of three behaviors performed by male flesh flies in our study, *bobbing, stilt,* and *jump* (for descriptions, see Table 1), is not understood. In the paired male experiments, *bobbing* showed a significant increase in frequency between days 1 and 3 but declined somewhat from this high level on days 4 and 6 (Fig. 9C). There were no significant transitions to or from *bobbing* on day 1 but there were at least two significant transitions (always including *bobbing–standing* and *grooming–bobbing*) for each day of age thereafter. In the single male experiments, *bobbing* was nonexistent, suggesting that this behavior may play some role in the interactions between flies. *Stilt*, in contrast, showed a significant decline in frequency with age in the paired male experiments (Fig. 9B), although it maintained at least two significant transitions with other behaviors at each day of age (always including *stilt–standing*). The age-related pattern for *stilt* was different in the single male experiments, occurring at low levels on day 1 and exhibiting a significant peak in frequency on day 3 (Fig. 13E). Occurrences of *jump* showed a significant increase between days 1 and 3 in the paired male experiments (Fig. 9A). In accord with the finding that *jump* rarely occurred in the single fly experiments, it appears that this behavior typically may occur in response to activity by or the presence of other flies. Neither *bobbing* nor *jump* ever participated in any significant transitions associated with aggressive behaviors but *stilt* served as a precedent for *low-intensity aggression* on days 2 and 4 and for *lunge* on day 2. Interestingly, *jump* was a precedent for *turn toward* on days 2 through 6 and followed *turn toward* on days 4 and 6, suggesting that it may occur as a reaction to another fly’s activity. *Jump*, in this study, is characterized as a vertical leap (Table 1) but may serve as the lift-off for flight (which is constrained in our test arena). Deeper insights into the possible role of these three behaviors in social encounters might be obtained through high resolution, simultaneous temporal analyses of the behaviors elicited by both members of the pair, with the goal of determining exactly what movements in one fly influence activity in the other. None of the three behaviors (*bobbing*, *stilt*, and *jump*) were noted in studies of male-male agonistic interactions in *D. melanogaster* [24] or *T. dalmanni* [18]. Both of these studies, however, were concerned only with behaviors performed during the aggressive encounters. Absent from our study but present in *D. melanogaster* [24] were observations of the behavior *wing threat*, classified as a low-intensity aggressive act. Also absent from our observations were two low-intensity aggressive behaviors performed by *T. dalmanni* [18]: *flex*, involving flexion and flicking of the forelegs in the direction of the opponent, and *line up eye stalks,* in which the two opponents confront each other face-to-face with their eye stalks aligned in parallel.

The flesh fly *S. crassipalpis* provides an intriguing comparative model system for the study of aggression because its natural lifestyle, including mating behavior, is very different from that observed in fruit flies and stalk-eyed flies. Our findings of fundamental differences in the organization of aggressive behaviors between the flesh fly and these other model systems are consistent with these lifestyle differences. Although our investigation of aggression in *S. crassipalpis* males shares some of the same analytical approaches used in the other model systems (e.g., behavioral transition matrices), it also adds two perspectives not previously explored in detail: the influences of age on the expression of aggressive behavior and the potential interactions of aggressive behaviors with other behaviors not performed during agonistic encounters. Extrapolating from our results, we maintain that such an approach will lead to a deeper understanding of the mechanisms controlling aggression.

## Supporting Information

File S1 **Behavioral transition matrices for the day 1, 2, 4, and 6 age cohorts in the paired male experiments.**
(DOC)

Video S1 **Exemplar of the behavior** ***lunge*** **.** Fly at the top lunges at its opponent.
(AVI)

Video S2 **Exemplars of the behaviors** ***lunge*** **and** ***hold*** **.** Fly to the right lunges and then holds its opponent; behaviors are shown at actual speed and ¼ speed.
(MPG)

Video S3 **Exemplars of the behaviors** ***hold*** **and** ***wrestle*** **.** Fly to the left approaches and then holds its opponent. This is followed quickly by wrestling, in which both flies are grasping and striking with their forelegs.
(AVI)

Video S4 **Exemplars of the behaviors** ***approach*** **and** ***avoid*** **.** Fly to the right approaches its opponent (standing). Then both flies avoid each other.
(AVI)

Video S5 **Exemplars of the behaviors** ***approach*** **and** ***retreat*** **.** Fly to the left approaches its opponent. Opponent then retreats to opposite side of the arena.
(AVI)
