# Correction: Extensive Reorganization of Behavior Accompanies Ontogeny of Aggression in Male Flesh Flies

**DOI:** 10.1371/journal.pone.0103919

**Published:** 2014-07-23

**Authors:** The PLOS ONE Staff

The following information is missing from the Funding section: National Science Foundation (1128954). The correct funding information is as follows: The authors thank the United States Department of Agriculture (2006-35302-7278), the Howard Hughes Medical Institute (52005872), and the National Science Foundation (1128954) for funding this project. The funders had no role in study design, data collection and analysis, decision to publish, or preparation of the manuscript.
