# Supplementary material for: Extensive Reorganization of Behavior Accompanies Ontogeny of Aggression in Male Flesh Flies
Source: PLoS One. 2014 Apr 8;9(4):e93196. doi: 10.1371/journal.pone.0093196 (PMC3979670; doi:10.1371/journal.pone.0093196)
Supplement: File S1 — Behavioral transition matrices for the day 1, 2, 4, and 6 age cohorts in the paired male experiments. (DOC) [file pone.0093196.s001.doc]

**File S1.** Behavioral transition matrices for the day 1, 2, 4, and 6 age cohorts.

The matrices summarize the frequencies at which each behavior (far left column) is followed by any other behavior (top row). Those transitions occurring more frequently than predicted by chance are indicated in bold. Descriptions of the behaviors (and their abbreviations) are summarized in Table 1.

Day 1.

|  | U | B | G | I | J | T | H | Wr | Av | W | Ap | Lo | L | R | Sti | | Sta | Σ |
| --- | --- | --- | --- | --- | --- | --- | --- | --- | --- | --- | --- | --- | --- | --- | --- | --- | --- | --- |
| U | 0 | 0 | 13 | 0 | 0 | 0 | 0 | **6** | 1 | **164** | 3 | 0 | 0 | 0 | 0 | **82** | | 269 |
| B | 0 | 0 | 0 | 0 | 0 | 0 | 0 | 0 | 0 | 1 | 0 | 0 | 0 | 0 | 0 | 1 | | 2 |
| G | 8 | 1 | 0 | 0 | 3 | 4 | 0 | 0 | 43 | 322 | 8 | 1 | 1 | 2 | 8 | **456** | | 857 |
| I | **2** | 0 | 0 | 0 | 0 | 0 | 0 | 0 | 0 | 0 | 0 | 0 | 0 | 0 | 0 | 0 | | 2 |
| J | 1 | 0 | 1 | 0 | 0 | 0 | 0 | 0 | 1 | **8** | 2 | 0 | 0 | 0 | 0 | 1 | | 14 |
| T | 0 | 0 | 2 | 0 | 0 | 0 | 0 | 0 | **8** | 9 | **10** | 1 | 0 | 0 | 0 | 3 | | 33 |
| H | **1** | 0 | 0 | 0 | 0 | 0 | 0 | 0 | 0 | 1 | 0 | 0 | 0 | 0 | 0 | 0 | | 2 |
| Wr | 0 | 0 | 0 | 0 | 0 | 0 | 0 | 0 | 0 | **7** | 0 | 0 | 0 | 0 | 0 | 1 | | 8 |
| Av | **55** | 0 | 14 | 0 | 2 | **6** | 0 | 1 | 0 | **268** | 41 | **17** | 0 | **14** | 7 | 167 | | 592 |
| W | **131** | 1 | **436** | 0 | 6 | 9 | 0 | 0 | 38 | 0 | **388** | 2 | 0 | 3 | 16 | 342 | | 1372 |
| Ap | 15 | 0 | 22 | 0 | 1 | 0 | 0 | 0 | **231** | 78 | 0 | **46** | **6** | **13** | 9 | 72 | | 493 |
| Lo | 5 | 0 | 2 | **2** | 0 | 0 | 0 | 0 | **32** | 10 | 9 | 0 | **2** | **4** | **4** | 17 | | 87 |
| L | 0 | 0 | 0 | 0 | 0 | 0 | **2** | 0 | 2 | **5** | 0 | **1** | 0 | 0 | 0 | 0 | | 10 |
| R | **7** | 0 | 0 | 0 | 0 | 0 | 0 | 0 | 6 | **21** | 1 | 0 | 0 | 0 | 0 | 11 | | 46 |
| Sti | 3 | 0 | 4 | 0 | 0 | 1 | 0 | 0 | **16** | 16 | 3 | 1 | 0 | 0 | 0 | **45** | | 89 |
| Sta | 40 | 0 | **368** | 0 | 2 | **13** | 0 | 1 | **215** | 459 | 27 | 18 | 1 | 10 | **45** | 0 | | 1199 |
| Σ | **268** | **2** | **862** | **2** | **14** | **33** | **2** | **8** | **593** | **1369** | **492** | **87** | **10** | **46** | **89** | **1198** | | 5075 |

Day 2.

|  | U | B | G | I | J | T | H | Wr | Av | W | Ap | Lo | L | R | Sti | Sta | Σ |
| --- | --- | --- | --- | --- | --- | --- | --- | --- | --- | --- | --- | --- | --- | --- | --- | --- | --- |
| U | 0 | 0 | 13 | **3** | 0 | 0 | **3** | **24** | 0 | **334** | 5 | 0 | **9** | 2 | 0 | 86 | 479 |
| B | 0 | 0 | 4 | 0 | 0 | 0 | 0 | 0 | 0 | 6 | 0 | 0 | 0 | 0 | 0 | **12** | 22 |
| G | 20 | **13** | 0 | 0 | 3 | 6 | 0 | 0 | 50 | 382 | 9 | 0 | 0 | 3 | 14 | **637** | 1137 |
| I | **5** | 0 | 0 | 0 | 0 | 0 | **1** | **3** | 0 | 8 | 0 | 0 | 0 | 0 | 0 | 1 | 18 |
| J | 0 | **1** | 0 | **1** | 0 | **1** | 0 | 0 | **2** | 3 | 0 | 0 | 0 | 0 | 0 | 4 | 12 |
| T | 1 | 0 | 1 | 0 | 0 | 0 | 0 | 0 | **7** | 8 | **6** | **1** | 0 | 0 | 0 | 2 | 26 |
| H | **3** | 0 | 0 | **1** | 0 | 0 | 0 | **3** | 0 | **10** | 0 | 0 | 0 | **1** | 0 | 1 | 19 |
| Wr | **7** | 0 | 1 | 0 | 0 | 0 | **1** | 0 | 0 | **32** | 0 | 0 | 0 | **2** | 0 | 5 | 48 |
| Av | **66** | **3** | 14 | 1 | **2** | **7** | 0 | 1 | 0 | **192** | 42 | **21** | 2 | **23** | **8** | 101 | 483 |
| W | 290 | 4 | **451** | 2 | 3 | 10 | 0 | 2 | 22 | 0 | **397** | 0 | 1 | 5 | 12 | 414 | 1613 |
| Ap | 28 | 0 | 16 | **7** | 1 | 1 | 1 | **7** | **214** | 108 | 0 | **20** | **5** | **23** | 6 | 76 | 513 |
| Lo | 3 | 0 | 3 | 0 | 0 | 0 | 0 | **2** | **18** | 7 | 4 | 0 | **6** | 0 | 0 | 18 | 61 |
| L | 3 | 0 | 0 | 0 | 0 | 0 | **13** | **4** | 0 | 6 | 0 | 0 | 0 | 1 | 0 | 1 | 28 |
| R | **15** | **1** | 0 | **1** | 0 | 0 | 0 | 1 | 3 | **46** | 3 | 0 | 0 | 0 | 0 | 10 | 80 |
| Sti | 5 | 0 | 0 | 0 | 0 | 0 | 0 | 0 | 5 | 13 | 5 | **2** | **1** | 1 | 0 | **28** | 60 |
| Sta | 27 | 0 | **637** | 2 | 3 | 1 | 0 | 1 | **163** | 455 | 42 | 17 | 4 | 19 | **20** | 0 | 1391 |
| Σ | 473 | 22 | 1140 | 18 | 12 | 26 | 19 | 48 | 484 | 1610 | 513 | 61 | 28 | 80 | 60 | 1396 | 5990 |

Day 4.

|  | U | B | G | I | J | T | H | Wr | Av | W | Ap | Lo | L | R | Sti | Sta | Σ |
| --- | --- | --- | --- | --- | --- | --- | --- | --- | --- | --- | --- | --- | --- | --- | --- | --- | --- |
| U | 0 | 0 | 41 | 6 | 0 | 0 | 3 | **62** | 0 | **424** | 4 | 0 | **11** | 1 | 0 | **169** | 721 |
| B | 1 | 0 | 10 | 0 | 0 | 0 | 0 | 0 | 1 | 0 | 0 | 0 | 0 | 0 | 0 | **18** | 30 |
| G | 34 | **23** | 0 | 1 | 7 | **14** | 0 | 0 | 24 | **412** | 12 | 1 | 1 | 7 | 6 | **355** | 897 |
| I | **10** | 0 | 0 | 0 | 0 | 0 | 0 | **27** | 0 | 10 | 0 | 0 | 0 | 1 | 0 | 0 | 48 |
| J | **6** | 0 | 0 | 0 | 0 | **4** | 0 | 0 | 2 | 7 | 0 | 0 | **1** | 0 | 0 | **10** | 30 |
| T | 0 | 0 | 3 | 0 | **1** | 0 | 0 | 0 | **4** | 6 | 3 | 0 | **10** | **3** | 0 | 8 | 38 |
| H | 8 | 0 | 0 | 0 | 0 | 0 | 0 | **27** | 0 | 11 | 0 | 0 | 0 | 1 | 0 | 1 | 48 |
| Wr | **48** | 0 | 0 | **23** | 0 | 0 | **23** | 0 | 0 | 41 | 0 | 0 | 1 | 4 | 0 | 14 | 154 |
| Av | **49** | 0 | 6 | 3 | 0 | 0 | 0 | 1 | 0 | 85 | 18 | **6** | 0 | **26** | 3 | 50 | 247 |
| W | **420** | 0 | **466** | 4 | 6 | 4 | 0 | 0 | 4 | 0 | **293** | 0 | 2 | 3 | 4 | 256 | 1462 |
| Ap | 37 | 0 | 18 | **7** | 1 | 0 | 0 | 7 | **118** | 63 | 0 | **6** | **11** | **34** | **4** | 44 | 350 |
| Lo | 1 | 0 | 0 | 0 | 0 | 0 | 0 | 0 | **4** | 2 | 1 | 0 | **2** | **2** | 0 | 5 | 17 |
| L | 1 | 0 | 0 | 0 | 0 | 0 | **21** | **27** | 0 | 0 | 0 | **1** | 0 | 0 | 0 | 3 | 53 |
| R | **36** | 0 | 1 | 1 | **3** | 1 | 0 | 3 | 1 | **49** | 1 | 0 | 2 | 0 | 0 | 13 | 111 |
| Sti | 4 | 0 | 5 | 0 | **1** | 0 | 0 | 0 | **4** | 2 | 2 | **1** | 0 | 0 | 0 | **15** | 34 |
| Sta | 60 | 7 | **356** | 2 | **11** | **15** | 0 | 0 | **87** | 349 | 16 | 2 | 12 | **29** | **17** | 0 | 963 |
| Σ | 715 | 30 | 906 | 47 | 30 | 38 | 47 | 154 | 249 | 1461 | 350 | 17 | 53 | 111 | 34 | 961 | 5203 |

Day 6.

|  | U | B | G | I | J | T | H | Wr | Av | W | Ap | Lo | L | R | Sti | Sta | Σ |
| --- | --- | --- | --- | --- | --- | --- | --- | --- | --- | --- | --- | --- | --- | --- | --- | --- | --- |
| U | 0 | 1 | 63 | 2 | 0 | 0 | 1 | **112** | 0 | **604** | 4 | 0 | **24** | 4 | 0 | **190** | 1005 |
| B | 1 | 0 | 8 | 0 | 0 | 0 | 0 | 0 | 0 | 6 | 0 | 0 | 0 | 0 | 0 | **23** | 38 |
| G | 40 | **16** | 0 | 0 | 14 | **13** | 0 | 1 | 47 | **628** | 17 | 3 | 4 | 22 | 4 | **383** | 1192 |
| I | 9 | 0 | 0 | 0 | 0 | 0 | 0 | **41** | 1 | 7 | 0 | **1** | 0 | 1 | 0 | 4 | 64 |
| J | 2 | 0 | 0 | 0 | 0 | **3** | 0 | 0 | 1 | 12 | 0 | 0 | 0 | 2 | 0 | **15** | 35 |
| T | 1 | 0 | 5 | 1 | **2** | 0 | 0 | 0 | **6** | 8 | **8** | 0 | **8** | **3** | 0 | 4 | 46 |
| H | **12** | 0 | 0 | 0 | 0 | 0 | 0 | **42** | 0 | 4 | 0 | 0 | 0 | 1 | 0 | 6 | 65 |
| Wr | **134** | 0 | 1 | **25** | 0 | 0 | **24** | 0 | 0 | 45 | 0 | 0 | 1 | **12** | 0 | 24 | 266 |
| Av | **78** | 0 | 14 | 2 | 0 | **9** | 1 | 1 | 0 | 145 | 22 | **6** | 4 | **29** | 1 | 48 | 360 |
| W | **571** | 0 | **660** | 9 | 3 | 5 | 1 | 5 | 25 | 0 | **499** | 0 | 4 | 20 | 6 | 280 | 2088 |
| Ap | 60 | 0 | 25 | **15** | 2 | 2 | 1 | 22 | **183** | 135 | 0 | **10** | **24** | **66** | 2 | 45 | 592 |
| Lo | 2 | 0 | 2 | **2** | 0 | 0 | **1** | 0 | **8** | 2 | 2 | 0 | **3** | 0 | 0 | 4 | 26 |
| L | 3 | 0 | 0 | 1 | 1 | 0 | **36** | **38** | 0 | 4 | 1 | 0 | 0 | 3 | 0 | 2 | 89 |
| R | 34 | 0 | 2 | 1 | 0 | 0 | 0 | 3 | 5 | **125** | 12 | 1 | 3 | 0 | 0 | **39** | 225 |
| Sti | 1 | 0 | 2 | 0 | 0 | **1** | 0 | 0 | 1 | 6 | 2 | 0 | 0 | 0 | 0 | **15** | 28 |
| Sta | 56 | **21** | **418** | 6 | **13** | **13** | 0 | 1 | **83** | 355 | 25 | 5 | 14 | **62** | **15** | 0 | 1087 |
| Σ | 1004 | 38 | 1200 | 64 | 35 | 46 | 65 | 266 | 360 | 2086 | 592 | 26 | 89 | 225 | 28 | 1082 | 7206 |
